# Cell-Type Specific Expression of the Vasopressin Gene Analyzed by AAV Mediated Gene Delivery of Promoter Deletion Constructs into the Rat SON In Vivo

**DOI:** 10.1371/journal.pone.0048860

**Published:** 2012-11-14

**Authors:** Todd A. Ponzio, Raymond L. Fields, Omar M. Rashid, Yasmmyn D. Salinas, Daniel Lubelski, Harold Gainer

**Affiliations:** Laboratory of Neurochemistry, National Institute of Neurological Disorders and Stroke, National Institutes of Health, Bethesda, Maryland, United States of America; UNC Eshelman School of Pharmacy, United States of America

## Abstract

The magnocellular neurons (MCNs) in the supraoptic nucleus (SON) of the hypothalamus selectively express either oxytocin (Oxt) or vasopressin (Avp) neuropeptide genes. In this paper we examine the cis-regulatory domains in the Avp gene promoter that are responsible for its cell-type specific expression. AAV vectors that contain various Avp gene promoter deletion constructs using EGFP as the reporter were stereotaxically injected into the rat SON. Two weeks following the injection immunohistochemical assays of EGFP expression from these constructs were done to determine whether the expressed EGFP reporter co-localizes with either the Oxt- or Avp-immunoreactivity in the MCNs. The results identify three major enhancer domains located at −2.0 to −1.5 kbp, −1.5 to −950 bp, and −950 to −543 bp in the Avp gene promoter that regulate the expression in Avp MCNs. The results also show that cell–type specific expression in Avp MCNs is maintained in constructs containing at least 288 bp of the promoter region upstream of the transcription start site, but this specificity is lost at 116 bp and below. Based on these data, we hypothesize that the −288 bp to −116 bp domain contains an Avp MCN specific activator and a possible repressor that inhibits expression in Oxt-MCNs, thereby leading to the cell-type specific expression of the Avp gene only in the Avp-MCNs.

## Introduction

Vasopressin is a neuropeptide well known for its classical functions of regulating blood pressure and systemic osmolality [1], [2], [3], and more recently, for its effects on social recognition, aggression, and other social behaviors [4], [5], [6], [7]. The magnocellular neurons (MCNs) in the supraoptic nucleus (SON) and paraventricular nucleus (PVN) of the hypothalamus selectively synthesize large quantities of either the oxytocin (Oxt) or vasopressin (Avp) neuropeptides which are then transported to the posterior pituitary where they are released into the blood stream [1]. The SON is a particularly good model for the study of cell-type specific expression of these neuropeptide genes since this nucleus contains primarily two distinct neuronal phenotypes, the Oxt and Avp synthesizing MCNs [8]. These MCNs are found in approximately equal numbers and selectively express either the Oxt or the Avp gene in about 97% of the MCN population in the SON. A small number of MCNs in the SON (about 3%) express both peptides at equivalent levels [9], [10], [11], [12]. At present, the mechanisms mediating the cell-type specific expression of Avp in the MCNs are unknown. Previous data from studies using transgenic rodents showed that −3.4 kb upstream of the transcription start site of the Avp gene was sufficient to produce cell-type specific gene expression of these peptides in the MCNs [13], [14], [15], [16].

In this study, we use stereotaxic microinjections of recombinant adeno-associated virus (rAAV) vectors carrying different promoter deletion constructs of the Avp gene into the rat supraoptic nucleus to dissect the upstream region of the Avp gene and to identify the cis-regulatory domains that are necessary for its cell-type specific expression. The various rAAV Avp gene promoter deletion constructs were fashioned to have EGFP as the reporter. Two weeks following injections of the rAAVs, immunohistochemical assays of EGFP expression from these constructs were done to determine whether the EGFP reporter co-localized with either the Oxt- or Avp-immunoreactivity (ir) in the MCNs. The results show that the key regions in the Avp gene promoter that regulate the cell-type specific expression in the SON are three distinct enhancer domains between −2.0 kb to −0.5 kb, an Avp MCN specific activator domain and a possible Oxt MCN specific repressor domain located between −288 bp to −116 bp upstream of the transcription start site (TSS). We hypothesize that the latter domain acts to direct expression selectively in Avp MCNs.

## Materials and Methods

### Animals

Adult male Sprague-Dawley rats were obtained from Charles River Laboratories (Wilmington MA, USA) and maintained under normal laboratory conditions (temperature: 21–23°C, 12 hour light-dark cycles with lights on at 6:00 AM) with access to food and water *ad libitum*. Rats were caged individually following surgical procedures and all animal procedures were performed in accordance with National Institutes of Health (NIH) guidelines on the care and use of animals, and according to a protocol (ASP # 1278) approved by the National Institute of Neurological Disorders and Stroke (NINDS) Animal Care and Use Committee.

### Plasmid Constructs

**Figure 1:**
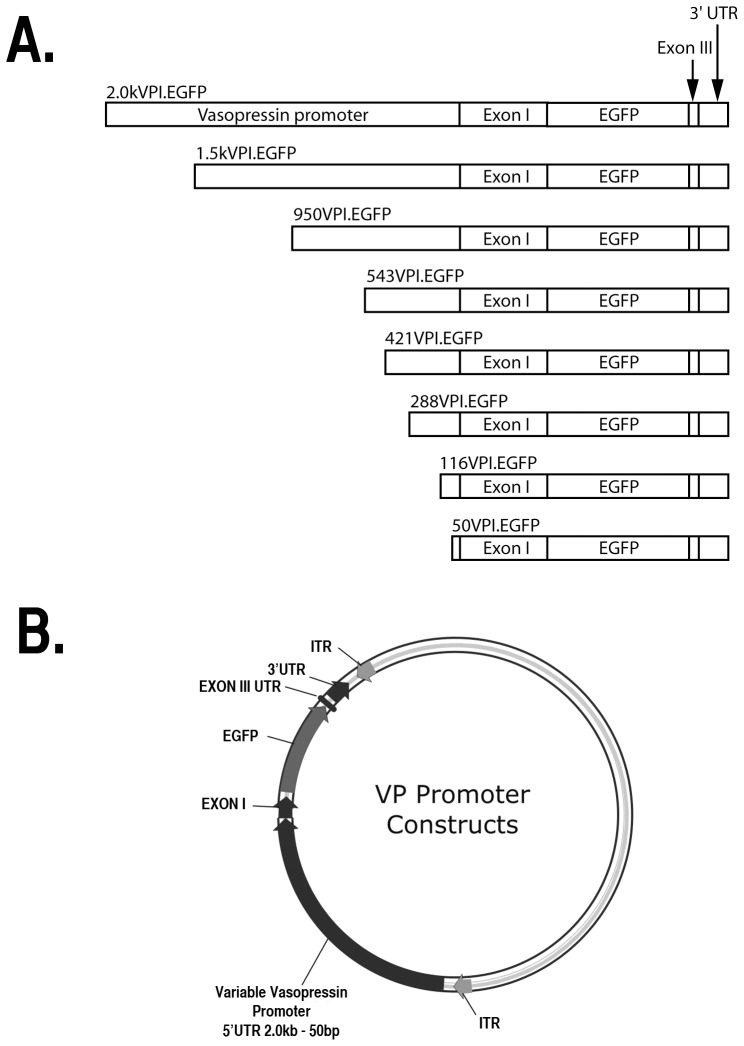
Mouse vasopressin deletion constructs and plasmid that were used to make the recombinant AAV (rAAV) vectors. A. All vasopressin constructs contained an EGFP reporter fused to exon I, as well as the endogenous poly-A signal found at the untranslated region of exon III, and an additional 173 bases that follow exon III. The vasopressin promoter region was systematically reduced such that promoter lengths tested were: 2.0 kbp, 1.5 kbp, 950 bp, 543 bp, 421 bp, 288 bp, 116 bp and 50 bp upstream of the transcription start site. B. Illustration of the AAV plasmid into which the assorted sequences described in A were inserted is shown. Abbreviations: ITR, inverted terminal repeat; 3′ UTR, 173 untranslated bases that follow exon III.

The pFBGR plasmid (a pFastBac™ variant obtained from Dr. Robert Kotin, NIH/NHLBI) was used to construct all the rAAV-Avp plasmids shown in Figure 1. The pFBGR contains two AAV inverted terminal repeats (ITRs) flanking the CMV promoter and EGFP reporter. Bacterial transposon Tn7 L and R attachment sites are located outside of the ITRs allowing the constructs to be incorporated into the shuttle bacmid bMON14272 found in DH10Bac™ E. Coli cells (Invitrogen, Carlsbad CA USA Cat# 10361-012). To make the longest Avp construct tested, a 2.0 kb fragment of mouse Avp promoter was amplified from plasmid 3.5VPIII.IGR2.1 [17] and ligated into VP-I-EGFP [18] to yield the 2.0 kVPI.EGFP insert (Figure 1). The pFBGR plasmid has XbaI sites just internal to the ITR sequences and the 2.0 kb and 1.5 kb VPI.EGFP inserts were made using XbaI-flanked PCR primers. All PCR reactions were done using the AccuPrime™ Polymerase System (Invitrogen, Carlsbad CA, USA Cat# 12339-016) according to the manufacturer's instructions and with a thermal cycler program of 94°C for 1 min, followed by 30 cycles of 94°C for 20 s, 57°C for 30 s, 68°C for 1 min/kb product. The 1.5 kbp, 950 bp, 543 bp, 421 bp, 288 bp, 116 bp, and 50 bp Avp promoter constructs shown in Figure 1 were made by excision of the upstream 5′ region via restriction enzyme digestion followed by re-ligation.

All the constructs in Figure 1 contained 173 bp of the Avp 3′UTR at their 3′ ends.

### rAAV construction and titers

We used a recombinant Baculovirus (rBV) method that employed insect cells for the production of rAAVs, which has been described elsewhere [19]–[23], [27]. Specific details related to the Avp constructs used in this paper are described in Methods S1.

AAV titers were determined by qPCR through comparison with a standard curve generated using pFBGR DNA and the following primers: EGFP F-ACCCTCGTGACCACCCTGAC and EGFP R-ACCTTGATGCCGTTCTTCTGC. PCR conditions were the same as above for the rBV titers and recombinant AAV titers were determined by comparison to a standard curve generated using pFBGR plasmid DNA as template and are expressed as viral genomes/ml (vg/ml). Final volumes of prepared rAAV were 20–100 µl. Final titers ranged from 2.0×10^12^–7×10^12^ vg/ml as determined by the above qPCR method.

### Stereotaxic targeting of rAAVs into the rat SON

**Figure 2:**
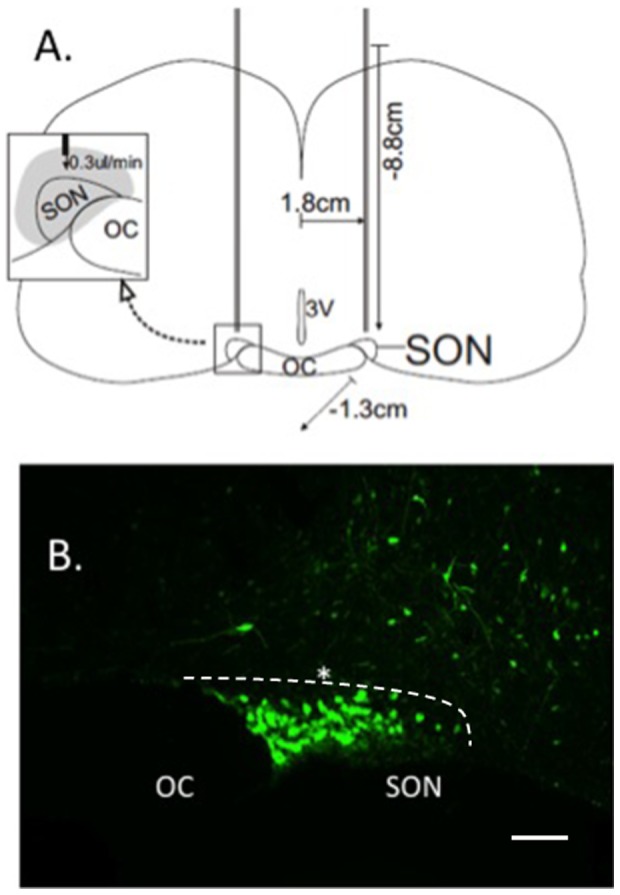
Stereotaxic microinjection of AAV vectors into the supraoptic nuclei (SONs) of adult male rats. A. Illustrates the coordinates of the injections relative to bregma: −1.3 mm caudal, 1.8 mm lateral (left and right), and −8.8 mm ventral. A total volume of 3 µl of vector was injected at a convection-enhanced delivery rate of 0.3 µl/minute, which resulted in vector completely filling the SON (see inset in A). B. Shows a view of a unilateral SON in a coronal section of the rat hypothalamus after injection into the SON of a rAAV containing the pan-specific CMV promoter fused to an EGFP reporter. Note that the MCNs in the SON show intense EGFP fluorescence and that areas dorsal to the SON also show significant although much less dense cellular fluorescence. This illustrates the total area of transduction deriving from this AAV injection. Asterisk shows the estimated position of the tip of the injection needle. The white dotted line borders the dorsal side of the SON. Scale line represents 100 µm. Abbreviations: OC, optic chiasm; SON, supraoptic nucleus.

1–2 month old rats (280–400 g) were anesthetized with 1–5% isoflurane (Baxter, Deerfield, IL USA) in a Stoelting gas anesthesia adaptor for stereotaxic instruments (David Kopf Instruments, Tujuna, CA, USA) and placed into a stereotaxic apparatus (Stoelting, Wood Dale, IL) in flat-skull position. The scalp of the rat was shaved and then sterilized with 100% betadine, 30% betadine/70% ethanol, and lastly 70% ethanol. A rostral-caudal incision was made to access the skull and two holes were drilled dorsal to the SON at coordinates 1.3 mm posterior to bregma; 1.8 mm lateral on each side as according to a stereotaxic atlas [24]. A 10 ul Hamilton Gastight syringe with a 30 gauge needle was placed −8.8 to −9.0 mm ventral to bregma, and 3 ul of a given rAAV viral construct was delivered to the SON area at a rate of 0.3 ul/min as shown in Figure 2A. Viral titers of the final injected rAAVs ranged between 2–7×10^12^ vg/ml. Following the injections, skull holes were filled with bone wax (Medline, Mundelein IL, USA Cat# 32020) and the incision was closed with interrupted sutures. Ketoprofen (Fort Dodge Animal Health, Fort Dodge IA, USA) was administered (5 mg/kg) intraperitoneally after surgeries. Figure 2A shows a coronal section of a rat brain in a diagram illustrating the method used for injections into the SON. Figure 2B illustrates the robust fluorescence in MCNs after injection over the SON of an AAV containing the pan-specific CMV promoter fused to an EGFP reporter.

### Salt loading

Systemic hyperosmotic stimulation of rats was done in several experiments to investigate transcriptional regulation of the viral constructs. For this purpose, following one week of recovery time, injected rats were salt loaded with 2% NaCl in their drinking water for an additional week. At the end of this two-week period, animals were euthanized for data analysis.

### Immunohistochemistry

All animals were euthanized by overdose of isoflurane anesthesia (Baxter, Deerfield, IL USA) two weeks post rAAV injection. Rats were then immediately transcardially perfused with 50–100 ml of phosphate-buffered saline (PBS) followed by 200–250 ml of fixative solution (4% paraformaldehyde in PBS, pH 7.4) at a perfusion rate of 5 ml per min. Following perfusion, the brains were removed and cryoprotected with 5% sucrose in 0.9% saline for 4 hr to overnight at 4°C, followed by 10% and 15% sucrose solutions prepared in 0.9% saline in a similar manner, and then temporarily stored at 4°C until use. Coronal sections (16 µm) were cut on a cryostat (Reichert-Jung 2800; Frigocut, Heidelberg Germany) and mounted onto coated slides (Fisher Scientific 12-550-19, Pittsburgh PA, USA). The sections were rinsed with PBS for 5 min and incubated in PBS containing 0.3% Triton-X for 5 min, washed 3 times in PBS, followed by incubation in primary antibodies. Mouse monoclonal antibodies PS38 (against Oxt-neurophysin) or PS41 (against Avp-neurophysin) were used at dilutions of 1∶200 or 1∶100 respectively in PBS [25], [26]. The polyclonal antibody against Avp-neurophysin, THR (prepared by Dr. Alan Robinson, UCLA School of Medicine, and supplied by the Pituitary Hormones and Antisera Center, Torrance, California), was used at a dilution of 1∶2000, and a polyclonal antibody against the GFP protein (Abcam, Cambridge UK, Cat# Ab-2090) was used at a dilution of 1∶1000. All were incubated overnight at 4°C. After incubation in primary antibodies, the slides were rinsed three times with PBS and the PS38 and PS41 slides were incubated in Alexa Flour® 594-conjugated donkey anti-mouse (Molecular Probes, Eugene OR, USA) secondary antibody (diluted 1∶500) to yield cytoplasmic red fluorescence. Alexa Flour® 488 (Molecular Probes) secondary antibody (diluted 1∶500) was used to yield cytoplasmic green fluorescence in the experiments using antibodies to GFP. After completion of the final PBS washes following secondary antibody immunohistochemistry, 2–3 drops of ProLong® Gold (Invitrogen, Carlsbad CA USA, Cat# P36930) were placed onto each slide, and then a cover slip was carefully positioned over the sections. All photomicrographic images were taken using an Eclipse E400 fluorescent microscope (Nikon, Melville NY, USA) equipped with a Retiga Ex*i* (QImaging, BC, Canada) CCD camera. All the immunohistochemical data shown in this paper are representative of data based on bilateral SON injections from at least 5 rats. Measurements of the numbers of either Avp- or Oxt-identified MCNs that showed colocalization of EGFP expressed from the AAV vectors injected into the SON, were made by visual superimposition and cell-to-cell matching of the EGFP-stained and specific neurophysin antibody- stained MCNs in the central region of the SON after the double-label immunostaining.

## Results

### An AAV vector-mediated gene transfer approach to study the expression of Avp promoter - EGFP reporter constructs in the rat SON

The paradigm that we have developed to study the cell-type specific expression in the SON of the various Avp gene promoter constructs shown in Figure 1A requires the delivery of an AAV vector that effectively transduces both Avp and Oxt MCNs. We accomplish this by stereotaxically injecting rAAVs over the SON (Fig. 2A) and then routinely waiting for two weeks following injection of the rAAVs before examining either the endogenous EGFP fluorescence or by EGFP immunofluorescence in the MCNs in the SON. Figure 2B illustrates the robust endogenous EGFP fluorescence in the MCNs in an SON found two weeks after injection of an AAV containing a CMV promoter driving the expression of the EGFP. In a previous paper, we showed that both the Avp and Oxt MCNs in the SON were equally transduced by injection of a CMV-EGFP containing rAAV [27]. In that study we compared the number of cells in the SON that expressed EGFP from the pan-specific rAAV-CMV-EGFP construct to those that contained both EGFP fluorescence and also were immunostained by the pan-specific neurophysin monoclonal antibody, PS45, which reacts with all the MCNs in the SON. We found that of the 2,499 EGFP-labeled MCNs in the injected SONs that were assayed, which represented 69.1% of the total MCNs in the injected SON, 97.7% were double labeled with PS45. Hence, we concluded that the efficiency of transduction with the CMV promoter was approximately 69% and that the AAV vector we stereotaxically injected in the SON effectively transduced both the Oxt and the Avp MCNs. Given this demonstration of the effective transduction of both Avp and Oxt MCNs in the SON by the AAV vector injection, we then turned to the AAVs containing the Avp promoter deletions shown in Figure 1A.

**Figure 3:**
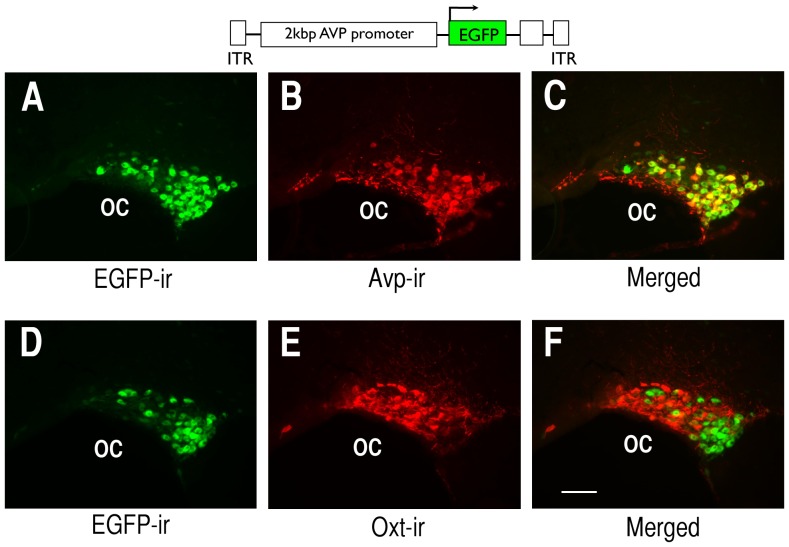
Cell-type specific expression of EGFP two weeks after injection of the 2.0 kbpVPI.EGFP rAAV in the SON. The endogenous fluorescence of the expressed EGFP (A, D) colocalizes with cells that are labeled by a vasopressin-neurophysin specific monoclonal antibody marker, PS41 (B), as seen in a merge of EGFP fluorescence and PS41 immunoreactivity (C), but does not colocalize with cells labeled with an oxytocin-neurophysin specific monoclonal antibody marker, (PS38) (E), as shown in the merged image in panel F. 100 µm scale bar in panel F is the same for all images. Abbreviation: OC, optic chiasm.

Our previous transgenic studies showed that a transgene containing 3.4 kb of the Avp promoter could produce robust cell-type specific gene expression in the rat SON [14]. However, this transgenic construct was 8.2 kbp in size and packaging this length of DNA into an AAV vector was not practical due to the size limitations for inserts into AAV [28]. In a previous study we found that much shorter Avp promoter lengths could produce expression in the SON in vitro [17], and unpublished data from the laboratory of Dr. Valery Grinevich indicated that a 2.0 kbp promoter in rAAV could produce cell-type specific expression in the SON in vivo (V. Grinevich, personal communication). Therefore, we decided to use a 2.0 kb Avp promoter for our initial promoter deletion experiments in vivo. Figure 3 illustrates the expression of the 2.0 kVPI.EGFP construct in Avp MCNs in the SON two weeks after injection of this AAV. Figure 3A shows the robust expression of endogenous EGFP fluorescence in the MCNs in a section showing the SON and the PS41-ir identifying the Avp MCNs in the same section is shown in Figure 3B. In the merged view in Figure 3C we can see many cells that are double labeled (yellow) but others that appear green. This apparent lack of colocalization in the green cells is due to the fact that cells that have more intense green fluorescence than their colocalized red fluorescence appear green in these unmanipulated images. Therefore, it is difficult to illustrate the full extent of colocalization by looking for exclusively yellow and no green cells in these untouched images. However, the lack of colocalization of the EGFP with the red Oxt specific MCN marker (PS38-ir) in the images shown in Figure 3D–F, which is not influenced by this photographic artifact, provides a more definitive test of cell-type specific gene expression. Figure 3D–F show another brain section from the same rat SON with the green fluorescence only in panel D, and the red PS38-ir (Oxt cell marker) staining shown in panel E. The merge is shown in panel F. In this case, the merged view in panel F shows that the green fluorescence (EGFP expression) is clearly occurring in MCNs that do not express the Oxt marker in red. These are the Avp MCNs since only the Avp and Oxt phenotypes exist in 97% of the MCNs in the SON. The near absence of yellow cells colocalized with PS38 and EGFP in panel F indicates the highly cell-specific expression of the 2.0 kVPI.EGFP construct in Avp MCNs in the SON.

In order to get a quantitative assessment of these colocalizations, in separate experiments we injected the 2.0 kbpVPI.EGFP into the SON and double label immunostained with either PS38, an Oxt–specific monoclonal antibody, or THR an Avp-specific neurophysin polyclonal antibody along with an EGFP polyclonal antibody (see Fig. S2). We then systematically evaluated the EGFP-expressing cells in the SON and compared them to their colocalized PS38 or THR immunoreactivities as described in Methods. The results showed that of 4979 EGFP expressing cells evaluated in the 2.0 kbp VPI-EGFP injected SONs, 4866 (or 97.7% %) were found to be colocalized in identified (THR-ir) Avp MCNs. In contrast, of 2288 EGFP-expressing MCNs counted in a second group of 2.0 kbp VPI-EGFP injected SONs, only 71 (or 2.6%) were found to be colocalized in identified (PS38-ir) Oxt MCNs. These quantitative data clearly correspond to the above conclusion about cell-type specific expression of the 2.0 kbp VP-EGFP construct which were drawn from interpretations of the images shown in Figure 3. The small 2.6% colocalization found between the EGFP-expressing green cells and the PS38-ir cells is comparable to the approximately 3% colocalization reported for the endogenous Oxt and Avp peptides in the SON [9], [10], [11], [12]. Therefore, we conclude that there is virtually no expression of the 2.0 kbp VPI-EGFP promoter construct in the Oxt MCNs in the SON. In addition, the 2.0 kbp VPI-EGFP promoter construct did not produce EGFP expression when injected into cortex (data not shown). These data indicate that the 2.0 kbp VPI-EGFP promoter construct produced excellent cell-type specific expression of the EGFP reporter in the SON, and we were reinforced in the view that we could use this AAV approach to study the other Avp promoter deletions shown in Figure 1A.

### Efficacy of expression of various Avp promoter deletion constructs in the SON

**Figure 4:**
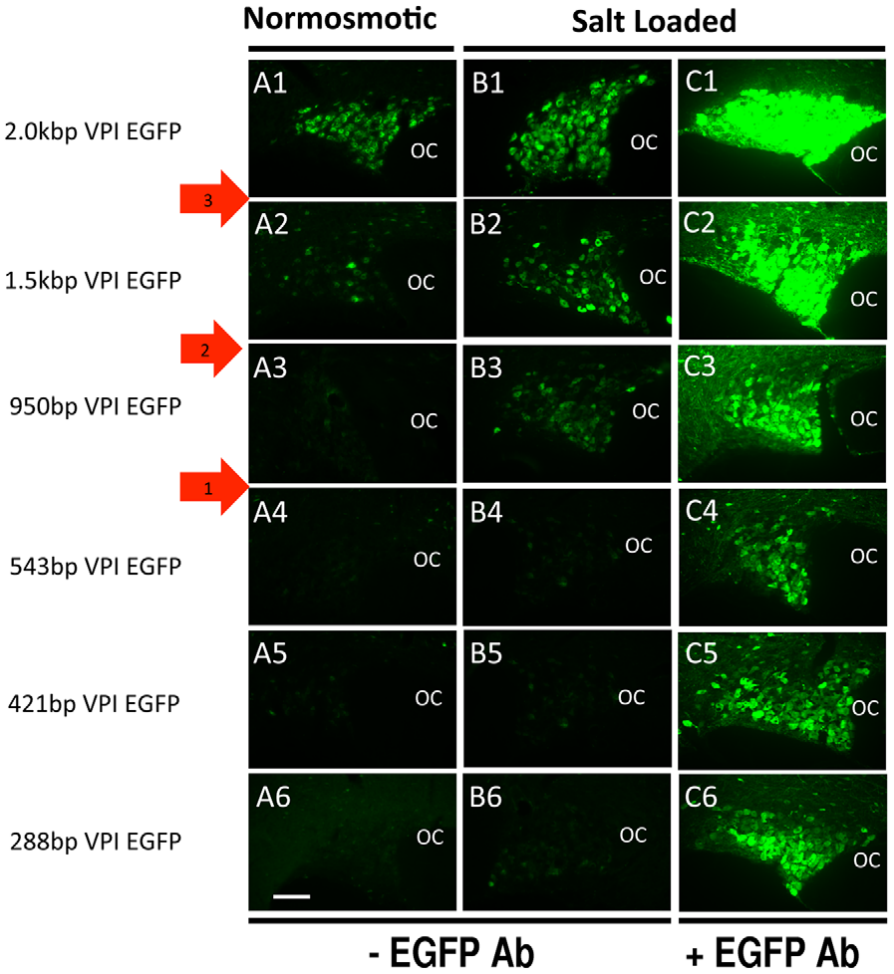
Efficacy of expression of the 2.0 kbp to 288 bp VPI.EGFP promoter deletion constructs (shown in **Fig. 1A**) in the SON. In these experiments after injection of the rAAVs into the SONs the control (normosmotic) rats were given water to drink for two weeks. In parallel experiments, the salt loaded rats were given water to drink for one week, followed by a second week of access only to water containing 2% NaCl (see Methods). The images in panels A1–6 and B1–6 present endogenous EGFP fluorescence (i.e. without the use of EGFP antibody in immunohistochemistry, −EGFP Ab). This was done in order to better assess quantitative differences between the constructs. In the third column (panels C1–6), IHC using EGFP antibodies together with salt loading was employed in order to maximize the detection of EGFP expression. Photographic image capture (exposure) times are identical for each panel. Under normosmotic conditions, clear expression of EGFP is obtained from injections of the 2.0 kVPI.EGFP (A1) and 1.5 kVPI.EGFP rAAV constructs (A2), and a much higher expression is seen for both under salt loading conditions (B1, B2). For the 950VPI.EGFP construct there was no detectable EGFP fluorescence under normosmotic conditions (A3), but salt loading was shown to significantly increase the EGFP expression in the SON (B3), and a greater increase in fluorescence was observed with both salt loading and IHC (C3). Neither normosmotic nor salt loading conditions produced detectable endogenous EGFP expression from the 543 bp to 288 bp VPI.EGFP constructs (A4–6, B4–6). However, application of both EGFP IHC (+EGFP Ab) and salt loading together clearly show that the EGFP was being expressed in the SON from the 543 bp to 288 bp VPI.EGFP constructs (C4–6). The red arrows denote three putative enhancer domains. Scale bar in panel A6 is 100 µm, and is the same for all the panels. Abbreviation: OC, optic chiasm.

Prior to studying the cell-type specific gene expression of the various promoter deletion constructs shown in Figure 1A, we first wanted to determine their relative abilities to produce EGFP expression after their packaging into AAVs and injection into the SON. The data shown in Figure 4 illustrate results for AAVs that contained promoter lengths ranging from −2.0 kbp to −288 bp upstream of the TSS. Since in this series of experiments we were focused on the efficacy of expression and not cell-type specific expression we initially assayed the endogenous EGFP fluorescence without IHC amplification, and these data are shown in columns A1–6 and B1–6. The data shown in Figure 4A1–6 were obtained from rats that were provided normal drinking water for the two weeks following the SON injections, and these rats are termed normosmotic. The rats whose SONs are illustrated in Figure 4B1–6 were osmotically stimulated in the second week after the SON injection by having only 2% saline to drink (termed salt loaded, see Methods). Salt loading is known to increase the expression of the endogenous Oxt and Avp genes in the SON [2] and was used here to see if the various promoter-EGFP constructs would be similarly affected. In the normosmotic rats the 2.0 kbp promoter construct produced a robust fluorescence (A1), the 1.5 kbp promoter construct produced considerably less fluorescence (A2), and the 950 bp promoter construct produced a barely detectable fluorescence (A3) in the SON. No fluorescence at all was detected with the 543 bp, 421 bp, and 288 bp constructs (A4–6). It should be noted here that the titers of the rAAVs injected in the experiments illustrated in Figure 4 ranged between 2.0×10^12^–7×10^12^ vg/ml, and that there was no correlation between the viral vector titer used for the injections and the level of EGFP expression. We explicitly considered this issue by doing injections of the 950 bpVP construct at 2×10^12^, 1×10^13^, and 4×10^13^ vg/ml with no discernable differences in expression. We also did injections of the 116 bp construct at 3.5×10^12^ and 3×10^13^ vg/ml and the 50 bp construct at 2×10^12^ and 4×10^13^ vg/ml with no noticeable differences in expression.

In the salt loaded rats, the endogenous EGFP fluorescence was increased for all three large promoter constructs (B1–B3), indicating that the 2.0 kbp, 1.5 kbp, and 950 bp promoter constructs were all responsive to the systemic osmotic stimulation in a manner similar to the endogenous Avp gene. In contrast, the 543 bp, 421 bp, and 288 bp constructs produced no detectable endogenous EGFP fluorescence, even after salt loading (B4–6). The progressive decreases in EGFP expression following these systematic deletions of the Avp promoter suggests that there may be three domains containing enhancer elements located in the 2.0 kb to 1.5 kb, 1.5 kb to 950 bp, and 950 bp to 543 bp regions upstream of the Avp transcription start site (see red arrows in Fig. 4). The promoter deletion constructs below 543 bp did not express sufficient endogenous EGFP fluorescence in either normosmotic or salt loaded rats to allow for an evaluation of possible enhancer sites in these regions. In fact, only for the 2.0 kbp construct was the endogenous EGFP fluorescence adequate for the cell-type expression assay (see Fig. 3). However, the use of an antibody against EGFP in an immunofluorescence assay did permit visualization of EGFP expression in the SON for all the constructs evaluated (Figs. 4C1–C6). The data in Figures 4C4–6 clearly demonstrate that the 543 bp, 421 bp, and 288 bp constructs were also effective in expressing EGFP in the MCNs, and that the EGFP immunofluorescence assay together with salt loading produced sufficiently vibrant EGFP labeling of the MCNs to allow for analysis of cell-type specific expression of all these constructs in the SON.

**Figure 5:**
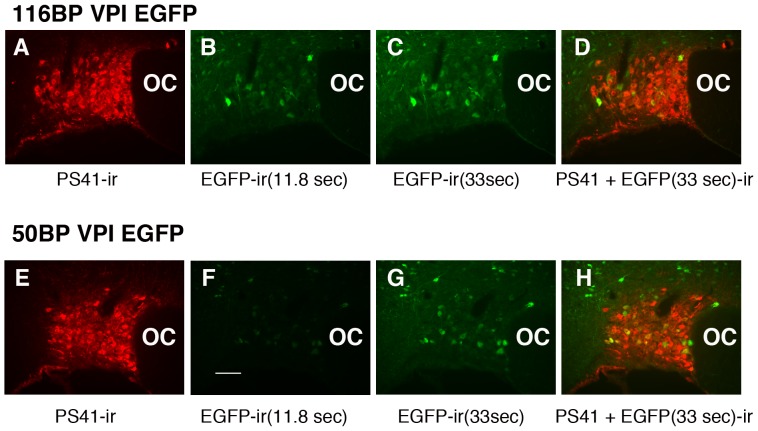
Efficacy of expression of the 116 bp (A–D) and 50 bp (E–H) VPI.EGFP promoter deletion constructs in the SON. In these experiments we found that both salt loading and IHC were required for any detection of the EGFP expression. In addition, exposure times needed to be increased three-fold from the 11.8 seconds used in Figure 4 (panels B and F), to 33 seconds (panels C and G) in order to achieve a level of detection of expression that would allow for double label analyses of cell-type specific expression (see Figs. 7 and 8). Panels D and H represent merges of panels A and C, and E and G, respectively. 100 µm scale bar in panel F is the same for all images. Abbreviation: OC, optic chiasm.

Figure 5 shows EGFP expression from the 116 bp and 50 bp Avp promoter deletion constructs (see Fig. 1A) injected into the SON of rats that were salt loaded and evaluated by immunofluorescence. Even at these most optimal conditions (salt loading plus EGFP enhancement via antibody), very low levels of EGFP fluorescence (expression) were observed with these constructs (Figs. 5B and 5F). It should be noted here that the images in Figures 5B and 5F were captured at 11.8 sec exposure time, identical to the images taken of the SONs shown in Figure 4. In an effort to improve the visualization of the expression in the 116 bp and 50 bp injected SONs the exposure time to capture these images was increased threefold to 33 sec (see Figs. 5C and 5G). EGFP expression from the 116 bp and 50 bp AAV constructs was still minimal in the SON but was clearly visible in a few MCNs after increasing exposure time to 33 sec (Figs. 5C and 5G). The merges of panels A and C, and panels E and G are illustrated in D and H, respectively. These data indicate that there was a large decrease in EGFP expression when the Avp promoter length was decreased from 288 bp to 116 bp, thereby suggesting that an additional putative enhancer element might be located within the −288 bp to −116 bp domain of the Avp promoter upstream of the TSS.

### Promoter deletion analysis of cell-type specific Avp gene expression in the SON

**Figure 6:**
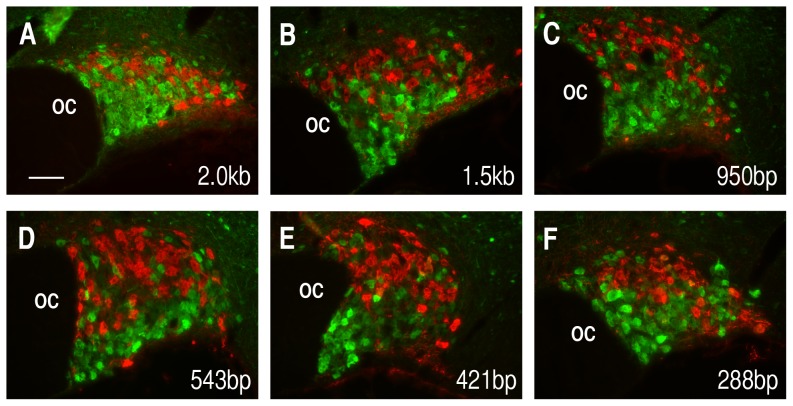
Analysis of the selectivity of expression of EGFP in MCNs from rAAVs containing the 2.0 kbp to 288 bp promoter deletion constructs (see **Fig. 1A**) two weeks after their injection into rat SONs. For each of the experiments the specific promoter deletion construct that was injected into the SONs is shown in the lower right of each panel. Immunohistochemical analyses confirm that each of the vasopressin rAAV constructs was preferentially expressed in vasopressin cells. EGFP immunoreactivity (in green) and oxytocin (PS38) immunoreactivity in red are shown as merged images in the panels. As a rule, the EGFP is not expressed in oxytocin immunoreactive cells. Occasionally EGFP was found to colocalize with oxytocin immunoreactivity to the extent of colocalization found for the endogenous peptide genes [9], [10]. While the data for the 2.0VPI.EGFP and 1.5VPI.EGFP constructs produce good endogenous EGFP fluorescence under normosmotic conditions (see Fig. 4), both salt loading as well as immunohistochemistry with antibodies to EGFP were required for optimal analysis of the 950–288 bp VPI.EGFP constructs. Scale bar in panel A is 100 µm, and is the same for all the panels. Abbreviations: OC, optic chiasm.

Having established the conditions necessary to visualize the EGFP expression for all of the AAV deletion constructs, double label IHC analysis was then performed to determine the locations of the regulatory elements in the Avp gene that might be responsible for its cell-type specific expression. Figure 6 shows typical results from injections of the Avp promoters ranging from 2.0 kbp to 288 bp. These are shown as merged views of PS38-ir (Oxt MCN marker) and EGFP-ir immunostained brain sections from SONs that had been injected with each deletion construct. The results illustrated in Figure 6 clearly show that all of the injected Avp constructs produced excellent cell-type specific expression even at the Avp promoter length of 288 bp. Therefore, we conclude that the cell-type specific elements in the Avp promoter are likely to be present within the 288 bp promoter.

**Figure 7:**
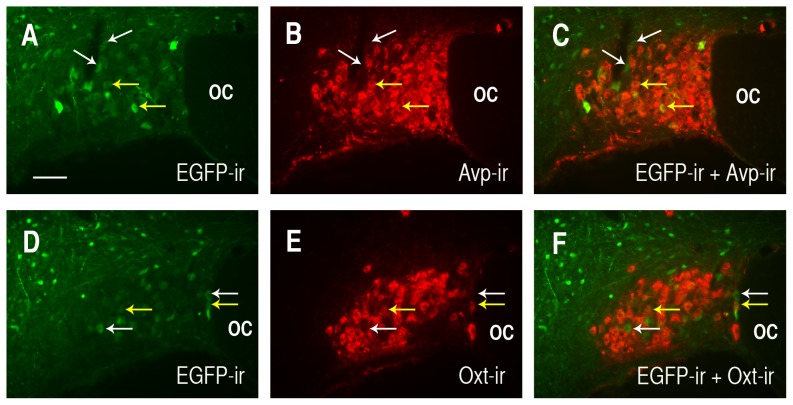
Analysis of the selectivity of expression of EGFP in MCNs in the SON after injections of rAAVs containing the 116 bp promoter deletion construct. Panels A and D illustrate the EGFP immunoreactivity (EGFP-ir) only, B shows the Avp marker (PS41-ir) only, E shows the Oxt marker (PS38-ir) only. The merged views of the EGFP-ir with either the Avp marker (PS41-ir in C) or the Oxt marker (PS38-ir in F) are shown. Examples of MCNs with colocalized EGFP-ir and PS38-ir or PS41-ir are depicted by yellow arrows and the white arrows show cells containing EGFP-ir only. The EGFP-ir was detected using 33 second photographic exposures as shown in Fig. 5. Scale bar in panel A is 100 µm, and is the same for all the panels. Abbreviations: OC, optic chiasm.

**Figure 8:**
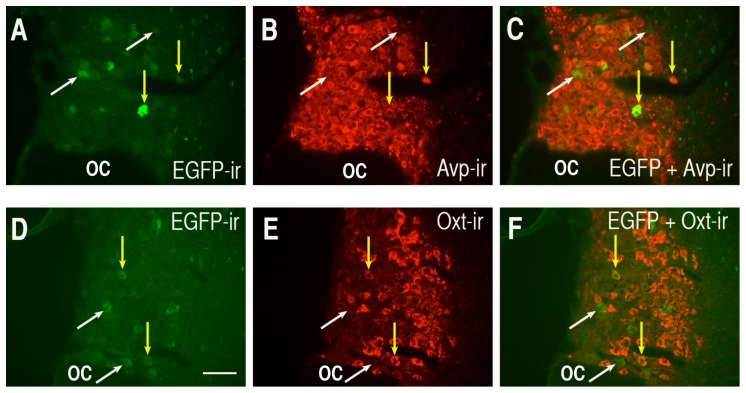
Analysis of the selectivity of expression of EGFP in MCNs in the SON after injections of rAAVs containing the 50 bp promoter deletion construct. Panels A and D illustrate the EGFP immunoreactivity (EGFP-ir) only, B shows the Avp marker (PS41-ir) only, E shows the Oxt marker (PS38) only. The merged views of the EGFP-ir with either the Avp marker (PS41-ir in C) or the Oxt marker (PS38-ir in F) are shown. Examples of MCNs with colocalized EGFP-ir and PS38-ir or PS41-ir are depicted by yellow arrows and the white arrows show cells containing EGFP-ir only. The EGFP-ir was detected using 33-second photographic exposures as shown in Fig. 5. Scale bar in panel D is 100 µm, and is the same for all the panels. Abbreviations: OC, optic chiasm.

The above data raise the question as to what is the lower cut-off length for the cell-type specific regulatory element in the Avp promoter. Figure 7 shows double labeling results following injection of a rAAV with an Avp promoter length of 116 bp. As seen in Figure 5 the EGFP expression from the 116 bp promoter construct was substantially weaker than the 288 bp construct (Figs. 4 and 6), even when a longer exposure image capture time of 33 seconds (Fig. 5C) was used. Since the red fluorescence of endogenous Avp-ir and Oxt-ir staining (Fig. 7, panels B and E, respectively) was so much more intense than colocalized EGFP fluorescence, the merged views of EGFP with PS41 (panel C) and EGFP with PS38 (panel F) rarely resulted in yellow cells. Therefore, we use yellow arrows in Figure 7 to indicate the EGFP-ir MCNs that were determined to be colocalized with either Avp-ir or Oxt-ir MCNs in the 116 bp injected SONs and white arrows to indicate MCNs that express EGFP and no peptide marker-ir. Similar observations were made using the 50 bp promoter construct and these results are depicted in Figure 8. Although it is relatively weak the green fluorescence observed in the cells transduced with either 116 bp AAVs (Fig. 7A) or 50 bp AAVs (Fig. 8A) are clearly greater in intensity than the absence of fluorescence observed when the same exposure times were used to photograph non-injected control tissues (see Fig. S1). Since the EGFP expression levels were low for both the 116 bp and 50 bp constructs we attempted to get quantitative assessments of the colocalizations of the EGFP-ir with the Avp (PS41-ir) or Oxt (PS38-ir) markers in the SONs after injection of the 116 and 50 AAV constructs. To do this we systematically evaluated the EGFP-expressing cells in the SON and compared them to their colocalization with either PS38 or PS41 immunoreactivities as was described in Methods (and used above for the 2.0 kbp construct injection). The results showed that of 376 EGFP-ir cells evaluated in the 116 bp VP-EGFP injected SONs, 292 (or 77.7%) were found colocalized in identified (PS41-ir) Avp MCNs, and of 210 EGFP-ir MCNs counted in a second group of 116 bp VP-EGFP injected SONs, 56 (or 26.7%) were found to be colocalized in identified (PS38-ir) Oxt MCNs. In the case of the 50 bp construct injected SONs, the results showed that of 265 EGFP-ir cells evaluated in the 50 bp VP-EGFP injected SONs, 157 (or 59.2% %) were found colocalized in identified (PS41-ir) Avp MCNs and of 210 EGFP-ir MCNs counted in a second group of 116 bp VP-EGFP injected SONs, 56 (or 26.7%) were found colocalized in identified (PS38-ir) Oxt MCNs. Therefore, these data indicate that the 116 bp and 50 bp lengths of the Avp promoter are markedly reduced in efficacy and selectivity of expression in the MCNs. The reduction of cell-type specificity of expression is suggested in that colocalization with the 50 bp and 116 bp Avp promoter constructs was about 30% in the Oxt MCNs, as opposed to the normal 2.6% found with the 2.0 kbp Avp-promoter. From these data we conclude that the cell-type specific regulatory element in the Avp promoter is located between −288 bp and −116 bp upstream of the TSS in the Avp gene.

### Osmotically regulated cis-domains in the Avp gene promoter

Vasopressin gene expression in rat MCNs is known to be regulated by changes in systemic osmolality [2]. We illustrate in Figure 4 that there were increases in expression of EGFP in the 2.0 kb, 1.5 kb, and 950 bp SON injected rats following salt loading (in Fig. 4, compare panels A1–A3 to panels B1–B3). Since there was no detectable endogenous EGFP fluorescence in Columns A4–5 and B4–5 in Figure 4, we could not determine if there was an osmotic response for the 543 bp and shorter promoter constructs. However, the fact that we could readily detect the EGFP expression by using the combination of salt loading and EGFP immunofluorescence, even for the shorter construct (288 bp) in Figure 4 suggested that we might be able to observe an osmotic effect if we reversed the order of the salt loading and the EGFP immunofluorescence assay. This experiment is illustrated in Figure S3 in which we applied the immunofluorescence assay to SONs in normosmotic rats that had been injected with the 288 bp promoter construct. By doing this, one can readily detect the EGFP fluorescence under control conditions (Fig. S3, panel B). Comparison of this control immunofluorescence in the SON in Figure S3, panel B to the immunofluorescence in the salt loaded SON (Fig. S3, panel C) clearly shows that the 288 bp promoter construct possesses an osmotically regulated element. Unfortunately, we were not able to extend this experiment to the 116 bp and 50 bp Avp promoter constructs, since salt loading and the EGFP immunofluorescence assay were both required in order to detect any EGFP fluorescence.

## Discussion

### AAV Strategy of cell-type specific gene expression

In the present study we examine which cis-element domains in the Avp gene promoter are responsible for cell-type specific regulation in the MCNs in the SON. In all mammalian species, the Avp gene which contains 3 exons and 2 introns, is located on the same chromosome as the closely related Oxt gene from which it is separated by a short intergenic region of as little as 3.5 kb in the mouse [29]. The Avp and Oxt genes are in a tail-to-tail arrangement and are transcribed towards each other from opposite strands of the DNA [2]. Given the structural similarities between the Oxt and Avp genes and their close apposition, the questions about what mechanisms are responsible for the highly selective, cell-type specific expression of these genes in the MCNs remains an intriguing and still unresolved fundamental question.

The most extensive and relevant experimental work that previously addressed this question came from studies of transgenic rodent models and the results of these studies are presented in reviews by Murphy and Wells [13], and Young and Gainer [16]. The consensus of these studies was that a transgene that contained <600 bp of the Oxt gene promoter region, or a transgene containing about 3. AVP gene, both 5 kbp 5′ upstream of the TSS, were sufficient to produce robust cell-type specific expression in MCNs in the hypothalamus. In these studies, in addition to the reporters, all of the introns and exons as well as various lengths of the 3′UTRs were present in the transgenes. In a previous report we proposed, based on evidence derived from biolistic transfections of hypothalamic organotypic cultures, that sequences in the 3′ UTR located downstream of exon III in both genes, specifically 178 bp in Avp and 432 bp in the Oxt gene, contributed to their cell-type specific expression in this in vitro model system [17]. However, definitive studies addressing whether any of these downstream elements (e.g., the exons, introns, and 3′ UTR) actually contributed to the cell-type specific expression of these genes in vivo were still needed. More recent experiments utilizing AAV gene delivery methods indicate that the gene bodies and downstream 3′ UTR are not essential for the cell-type specific expression in the MCNs in vivo [27], [30] (V Grinevich, personal communication).

Viral vector methods have been shown to be an effective means by which to deliver genes into the central nervous system in vivo [28], [31], [32], [33], [34], including for gene transfer into hypothalamic neurons [34], [35], [36], [37], [38] and specifically in MCNs [35], [36], [39], [40]. In addition, viral vectors have been successfully used to study gene promoter domains that are involved in cell-specific gene expression in vivo [27], [38], [41], [42], [43], [44] and AAV vectors have been shown to be more efficient than lentiviral vectors in transducing neurons in the forebrain [38], [40]. In previous studies it was shown that AAV vectors that contained promoter deletion constructs of the Oxt gene promoter fused to EGFP reporters effectively transduced MCNs in the rat SON in vivo [27], [30]. The results of these studies also demonstrated that the downstream components (exons, introns, and 3′ UTR) in the Oxt transgene were not necessary for cell-type specific expression in Oxt MCNs in vivo, and that the cell-type specific expression of the Oxt gene was due to elements found located in a −216 to −100 bp domain upstream of the TSS [27].

### Localization of the domains in the Avp gene promoter that regulate its cell-type specific expression

In this paper we use the highly efficient recombinant AAV method of gene transfer in vivo for the study of promoter deletion constructs of the Avp gene. Injections of AAVs containing 2.0 kbp of the DNA sequence upstream of the TSS in the Avp gene were found to produce robust expression of EGFP selectively in Avp- but not in the Oxt–MCNs (Fig. 3). Further experiments showed that AAV vectors containing 1.5 kbp, 950 bp, 543 bp, 421 bp and 228 bp (but not 50 bp or 116 bp) upstream sequences of the Avp gene promoter also could support cell-type specific gene expression in Avp MCNs (Fig. 6). However, the latter AAV constructs all required amplification either by salt loading or by EGFP antibody staining or both due to their lower levels of expression compared to the 2.0 kbp construct. In contrast, the intrinsic fluorescence from the 2.0 kbp VPI.EGFP AAV was sufficiently robust to be clearly observed in normosmotic rats either without salt loading or immunohistochemical amplification (Fig. 3). For this reason we suggest that there is a very strong enhancer domain located between −2.0 and −1.5 kb upstream of the Avp transcription start site. Comparisons between columns A and B in Figure 4 also suggest that there are at least two other enhancer sites between −1.5 kbp and −543 bp (see red arrows in Fig. 4) in the Avp promoter. Figure 5 shows that the AAVs containing the −50 bp and −116 bp upstream regions can also produce EGFP expression in the SON, but very weak and relatively non-selective expression in the MCNs (e.g., compare the selective expression produced by the 288 bp construct in Figure 6F to the results shown for 116 bp and 50 bp in Figures 7 and 8, respectively). One possibility to increase the expression efficacies of our Avp promoter constructs without influencing their cell-type specificities would be to link our promoter constructs with the recombinase Cre to drive EGFP flanked by loxP sites. This, in principle, could enhance the expression efficacy of the EGFP by the injections of AAVs containing the 116 bp and 50 bp Avp promoters that express Cre in order to drive EGFP expression from coinjected FLEX-GFP or flox-stop GFP vectors (or in a transgenic rodent line containing a Cre-activatable GFP). An especially valuable use of this approach in future physiological studies would be to link one of the more robustly expressing Avp promoters (e.g., the 2.0 kbpVPI.EGFP construct) to Cre to selectively knockout specific and physiologically interesting genes flanked by loxP sites selectively in Avp MCNs. All of the constructs that we injected did not contain introns 1 and 2 and exons 2 and 3 in the Avp gene (Fig. 1A), and therefore we conclude that these structures are not needed for cell-type specific expression in the Avp MCNs. In this regard, it should be noted that it has recently been shown that neither exon 1 nor the endogenous 3′ UTR which are present in our constructs are necessary for the robust and cell-type specific expression of the 2.0 kbp promoter construct (V. Grinevich, personal communication).

**Figure 9:**
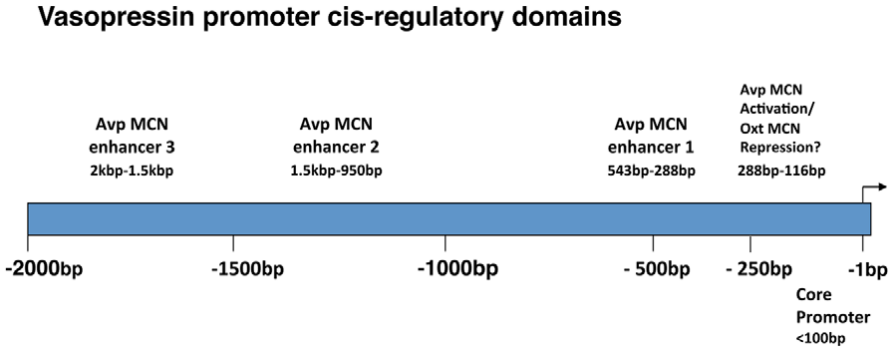
Diagram illustrating the cis-regulatory domains in the vasopressin gene promoter as suggested by the data in this study. Since the 288 VPI.EGFP construct still maintains the vasopressin MCN specific expression (Fig. 6F) we hypothesize that there is an activator of expression in the Avp MCNs and a possible repressor element with an upper boundary of −288 kbp that inhibits expression selectively in the Oxt MCNs. We further propose that there are at least three enhancer domains upstream of the transcription start site of the Avp gene that are involved in expression in the Avp MCNs, which we designate enhancer 3 located between −2 kbp to −1.5 kbp, enhancer 2 located between −1.5 kbp to −950 bp, and enhancer 1 located between −543 bp to −288 bp (see Fig. 4). The −288 to 116 bp region appears to contain the cell-type specific element(s) (see text), and an osmotically-responsive element also appears to be present within the 288 bp construct (see Fig. S3).

In summary, our promoter deletion experiments with the Avp gene show that AAV vectors containing 2.0 kbp, 1.5 kbp, 950 bp, 543 bp, 421 bp, and 288 bp upstream sequences of the Avp gene promoter can all support cell-type specific Avp gene expression in Avp-MCNs with no expression in Oxt MCNs (Fig. 6). Since the 116 bp and 50 kbp constructs produced very poor and nonselective expression in the MCNs, this strongly indicated that the domain between −288 and −116 bp must contain the regulatory elements that confer the cell-type specific expression. Interestingly, this is consistent with data from earlier experiments obtained by biolistic transfection of hypothalamic organotypic cultures which found that 288 bp promoter driven EGFP constructs could produce expression in Avp MCNs in vitro [27]. A summary of the regulatory domains in the Avp promoter which are responsible for the cell-type specific expression of the Avp gene are illustrated in Figure 9 which also proposes that the cell-type specific expression of the Avp gene primarily relys on multiple enhancers in the promoter of the Avp gene.

### Comparison of Avp promoter deletion data and previous transcription factor binding site predictions

In the 1990s a popular approach to identify regulatory elements that might be involved in the expression of genes was to first identify the evolutionarily conserved DNA sequences in the genes. For the Avp gene it had been noted that the highest sequence conservation was located in the non-coding DNA about 500 bps 5′ upstream of the TSS, thereby suggesting that significant regulatory sequences might be located in this domain [29], [45], [46], [47]. Therefore, transcription factor (TF) prediction programs were applied to the sequences that were 5′ upstream of the TSS in the Avp gene with the purpose of identifying putative Transcription Factor Binding Site (TFBS) motifs for further experimental study [45]. Many experiments to validate these TF and TFBS predictions were performed in heterologous tissue culture models in vitro and showed that many of these were potentially functional regulatory motifs in the Avp gene promoter [2]. These include one or more AP2 sites between −84 bp to 146 bp [45], AP1 sites at between −134 to −128 bp [48], [49], CRE between −227 and −116 bp [45], [50], [51], E box between −155 to −1341 bp [48], [52], [53], and SP 1 between −195 to −68 bp [48]. Interestingly, all of these which were predicted and in many cases functionally validated in vitro in the Avp promoter were located within the −288 to −116 bp domain that we propose contain the cell-type specific regulatory elements in the Avp gene (Fig. 9). However, experimental evidence for in vivo function is still largely lacking for most of these predicted motifs. Only for the E box motif has functional relevance been demonstrated in vivo, and in this case to regulate the circadian expression of Avp expression in the suprachiasmatic nucleus (SCN) [52]. Most interesting is that to our knowledge there has been only one motif predicted to be in the 2.0 kbp to 543 bp region in which the promoter deletion studies in this paper indicate have the most powerful enhancer activities for cell-type specific expression of the Avp gene in the MCNs. The predicted motif is the glucocorticoid responsive element (GRE) which is located at −622 to −608 bp in the Avp gene [45], [50]. However, this GRE motif is usually believed to act as a repressor of Avp expression [50] in the CRH neurons in the paraventricular nucleus (PVN), and is not believed to function in the MCNs in the PVN or SON [2]. Clearly, more functional studies must be done in vivo to reexamine the putative TFs and TFBSs in the Avp gene promoter responsible for the cell-type specific expression in the MCNs, and it is likely that more selective deletions of these putative sites together with the AAV gene transfer strategy described in this paper could be useful to accomplish this aim.

### Localization of the osmotically responsive element in the Avp promoter

The Oxt- and Avp-MCNs and their respective peptide genes are known to be highly regulated by systemic osmotic perturbations, e.g., salt loading and dehydration [2]. In our previous deletion study of the Oxt gene promoter using an AAV strategy we were surprised to find that the osmotic regulation of the Oxt gene was present in all of the deletion constructs studied, including the smallest −50 bp promoter construct [27]. This implied that the osmotic regulation in the Oxt gene resided in the core promoter domain, possibly at the Pol II binding site itself. In this paper, we were able to demonstrate that the osmotic regulation was present in the 288 bp promoter construct Avp gene (Fig. S3). We could obtain detectable EGFP expression with the 116 bp and 50 bp Avp promoter constructs only by using a combination of salt loading and immunofluorescence, and therefore we could not determine if the osmotic responsiveness extended below 288 bp. Although we could not perform meaningful osmotic perturbation experiments below 288 bp, the data in this study were sufficient to evaluate the conclusions of the only other papers in the literature that studied the osmotic responsiveness of the Avp gene. In those papers, heterologous cell lines (SCLC, HeLa, and HEK293) were transfected with promoter deletion constructs of the Avp gene and the authors reported osmotic regulatory sites that are located in the 5′ flanking sequence between −1500 and −532 bp upstream of the TSS in the Avp gene [54], [55]. While it is possible that the Avp gene promoter may exhibit such distal osmotically responsive elements in vitro, our in vivo data strongly suggest that the physiologically relevant site in the Avp promoter is at or below −288 bp.

The activations of the Avp and Oxt genes in response to systemic osmotic perturbations are not unique properties of these genes, and changes in expression in response to dehydration or salt loading can be found for many other endogenous genes in the MCNs. [56], [57]. In this regard, we wondered whether a completely unrelated exogenous gene inserted in the MCNs would show this response to systemic osmotic perturbations. Therefore, we injected rAAV constructs containing a CMV promoter linked to an EGFP reporter into SONs in normosmotic and salt loaded rats and compared the endogenous EGFP expression in these SONs (Fig. S4). As can be seen in Figure S4 there was a dramatic increase in EGFP expression in the MCNs in the SON following the salt loading procedure, and an equally dramatic axonal transport of the EGFP fluorescence to the neural lobe of the pituitary. Avp and Oxt promoter driven gene expression as well as cell volumes are known to change in response to systemic osmotic changes only in the MCNs [2], [58], [59]. Interestingly, other but not all of the genes that reside in the MCNs respond with changes in expression to osmotic perturbations [56], [60]. It would be interesting to know for all the genes that have been shown in microarray studies to be osmoregulated in vivo in the SON [56], [60], which DNA sequences in their promoters (including in the CMV promoter) might be associated with this global response to systemic osmotic perturbations.

### Summary & Conclusions

Based on data presented in this paper, we hypothesize that: 1) there is an Avp activator element (and possibly a repressor element to prevent expression in the Oxt-MCNs) in the −288 to −116 5′ upstream region of the Avp gene that regulates its cell-type specific expression in Avp-MCNs, and 2) that there are multiple enhancer elements in the 2.0 kbp to 543 bp region upstream of the TSS in the Avp gene which regulate expression in the Avp-MCNs in the SON. Another conclusion that can be clearly drawn from these studies is that the viral vector approach described herein is a highly effective method to experimentally study cell-type specific gene expression in the central nervous system, and could easily be applied to any gene and brain region of interest. In preliminary experiments we tested whether the AAV for our most powerful construct, the 2.0 kbp promoter, could produce expression in the Avp expressing neurons in the SCN. After injection of this AAV in the SCN, we could detect no EGFP expression in this nucleus. Clearly, other regulatory domains not present in our 2.0 kbp construct are necessary for basal expression in the SCN. In this regard, it should be noted that the vasopressin-EGFP transgenic rat in which the transgene contains the entire Avp structural gene flanked by 3 kbp of 5′upstream sequence and 2 kbp of 3′ downstream sequence, in addition to the EGFP reporter, was reported to express EGFP in the SCN [61]. Unfortunately, the size of this transgenic construct exceeds the insert size limit of the AAV vector. However, there are several other viral vectors other than AAV that can accept this insert size and even larger inserts [28], that could be used for promoter deletion studies comparable to those described in this paper. Therefore, by using alternative viral vectors it should be possible to determine which regulatory elements in the Avp gene, other than those identified in this study for cell-type specific Avp gene expression in the MCNs in the SON, will be needed to produce expression in other Avp expressing regions such as the SCN, parvocellular neurons in the PVN, Bed Nucleus of the Stria Terminalis (BNST) or the amygdala.

## Supporting Information

Figure S1 **Illustration of the control immunofluorescence present in a non-injected tissue section photographed using the same exposure time (33 second exposures) and magnification used for the photomicrographs shown in** **Figure 7** **and** **Figure 8** **.** Abbreviation : OC represents location of optic chiasm. Scale bar = 100 µm.
(TIF)

Figure S2 **Cell type specific expression of EGFP two weeks after injection of the 2.0 kbpVPI.EGFP rAAV in the SON.** The endogenous fluorescence of the expressed EGFP (A, D) colocalizes with cells that are labeled by a vasopressin-neurophysin specific rabbit polyclonal antibody marker, THR (B), as seen in a merge of EGFP fluorescence and THR immunoreactivity (C), but does not colocalize with cells labeled with an oxytocin-neurophysin specific mouse monoclonal antibody, marker, PS38) (E), as shown in the merged image (F). 100 µm scale bar in panel A is the same for all images. Abbreviation: OC, optic chiasm.
(TIF)

Figure S3 **Expression of the injected 288 bp VPI-EGFP construct in the MCNs in the SON is greatly increased during salt loading stimulation.** A. Endogenous EGFP fluorescence is not detectable in the SON under normosmotic conditions. B. EGFP expression is detectable in the SON under normosmotic conditions after EGFP IHC. C. EGFP- is strongly expressed in the SON under salt loading conditions with EGFP IHC illustrating that the element responsive to osmotic stimulation is present in the 288 bp promoter construct. 100 µm scale bar in panel C is the same for all images. Abbreviation: OC, optic chiasm.
(TIF)

Figure S4 **Expression of the cytomegalovirus (CMV) promoter driven expression of EGFP is dramatically increased under salt loading conditions.** Rats were injected with the construct shown in the top panel and, after one week, either maintained under control conditions (normosmotic) or subjected to salt loading for an additional week. Middle panels: Endogenous EGFP fluorescence (expression) in salt loaded SONs is much higher than in control (normosmotic) animals as illustrated in two separate rat brain sections. Consistent with this, robust EGFP fluorescence was also observed in the neural lobe of the pituitary gland (bottom panel) with salt loading. Scale bar = 100 µm. Abbreviation: OC, optic chiasm.
(TIF)

Methods S1 **Supplemental Methods.**
(DOCX)
