# Supplementary figures and images for: Cell-Type Specific Expression of the Vasopressin Gene Analyzed by AAV Mediated Gene Delivery of Promoter Deletion Constructs into the Rat SON In Vivo
Source: PLoS One. 2012 Nov 14;7(11):e48860. doi: 10.1371/journal.pone.0048860 (PMC3498266; doi:10.1371/journal.pone.0048860)

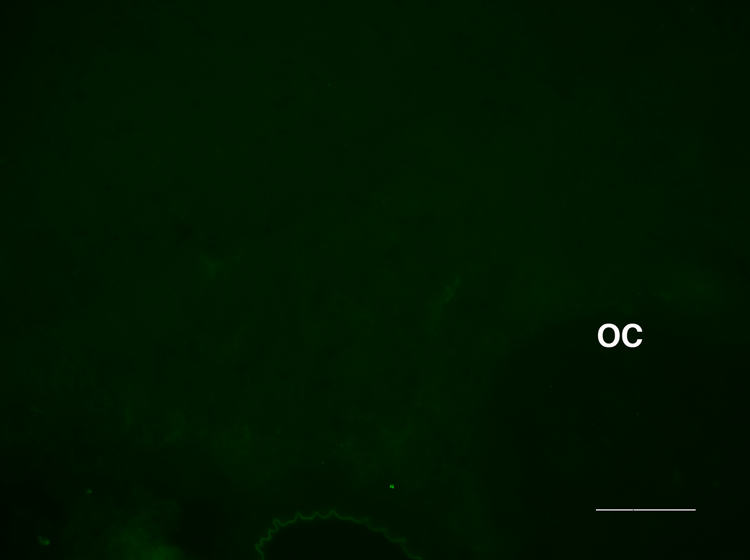

Supplement: Figure S1 — Illustration of the control immunofluorescence present in a non-injected tissue section photographed using the same exposure time (33 second exposures) and magnification used for the photomicrographs shown in Figure 7 and Figure 8 . Abbreviation : OC represents location of optic chiasm. Scale bar = 100 µm. (TIF) [file pone.0048860.s001.tif]

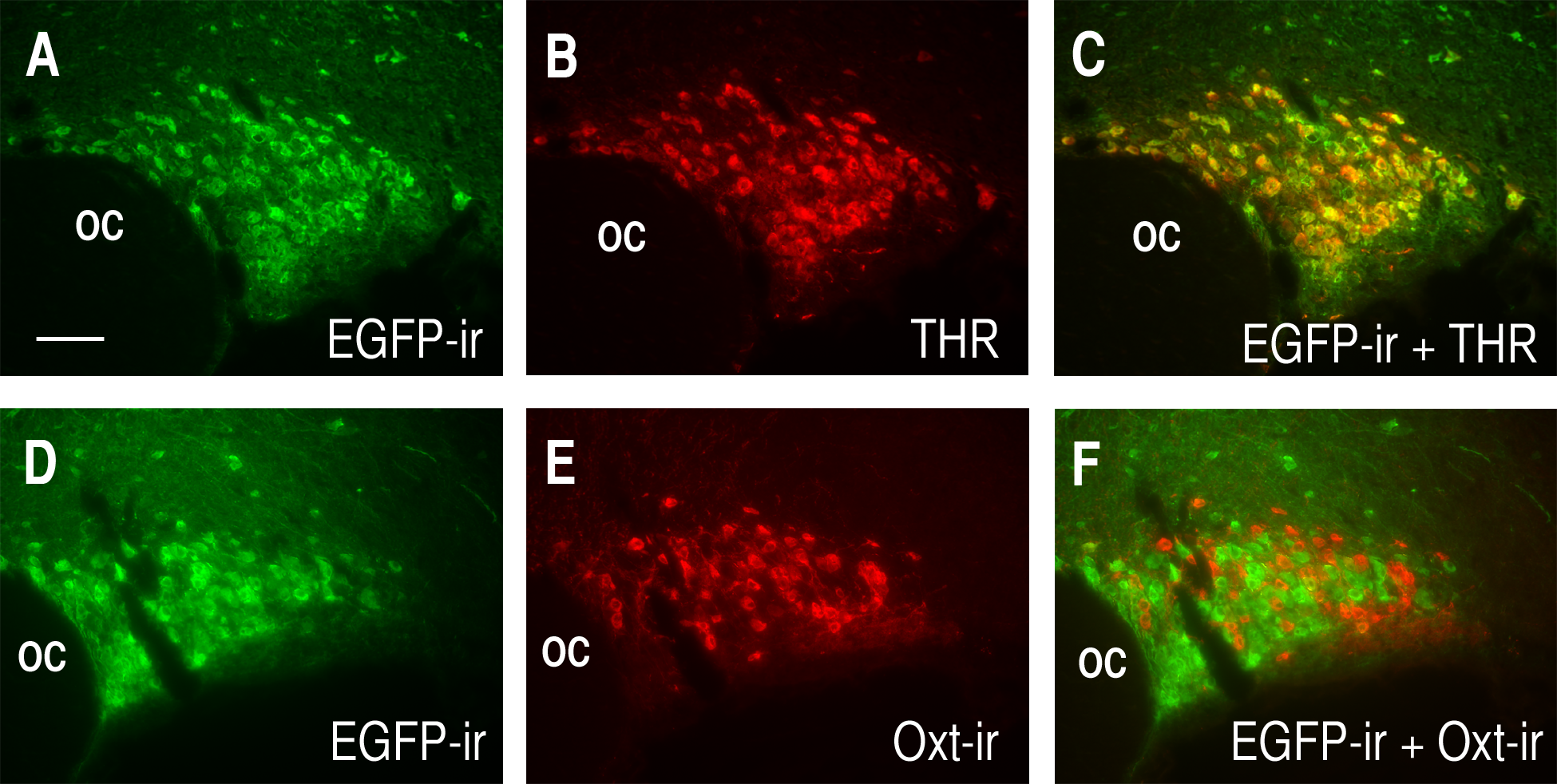

Supplement: Figure S2 — Cell type specific expression of EGFP two weeks after injection of the 2.0 kbpVPI.EGFP rAAV in the SON. The endogenous fluorescence of the expressed EGFP (A, D) colocalizes with cells that are labeled by a vasopressin-neurophysin specific rabbit polyclonal antibody marker, THR (B), as seen in a merge of EGFP fluorescence and THR immunoreactivity (C), but does not colocalize with cells labeled with an oxytocin-neurophysin specific mouse monoclonal antibody, marker, PS38) (E), as shown in the merged image (F). 100 µm scale bar in panel A is the same for all images. Abbreviation: OC, optic chiasm. (TIF) [file pone.0048860.s002.tif]

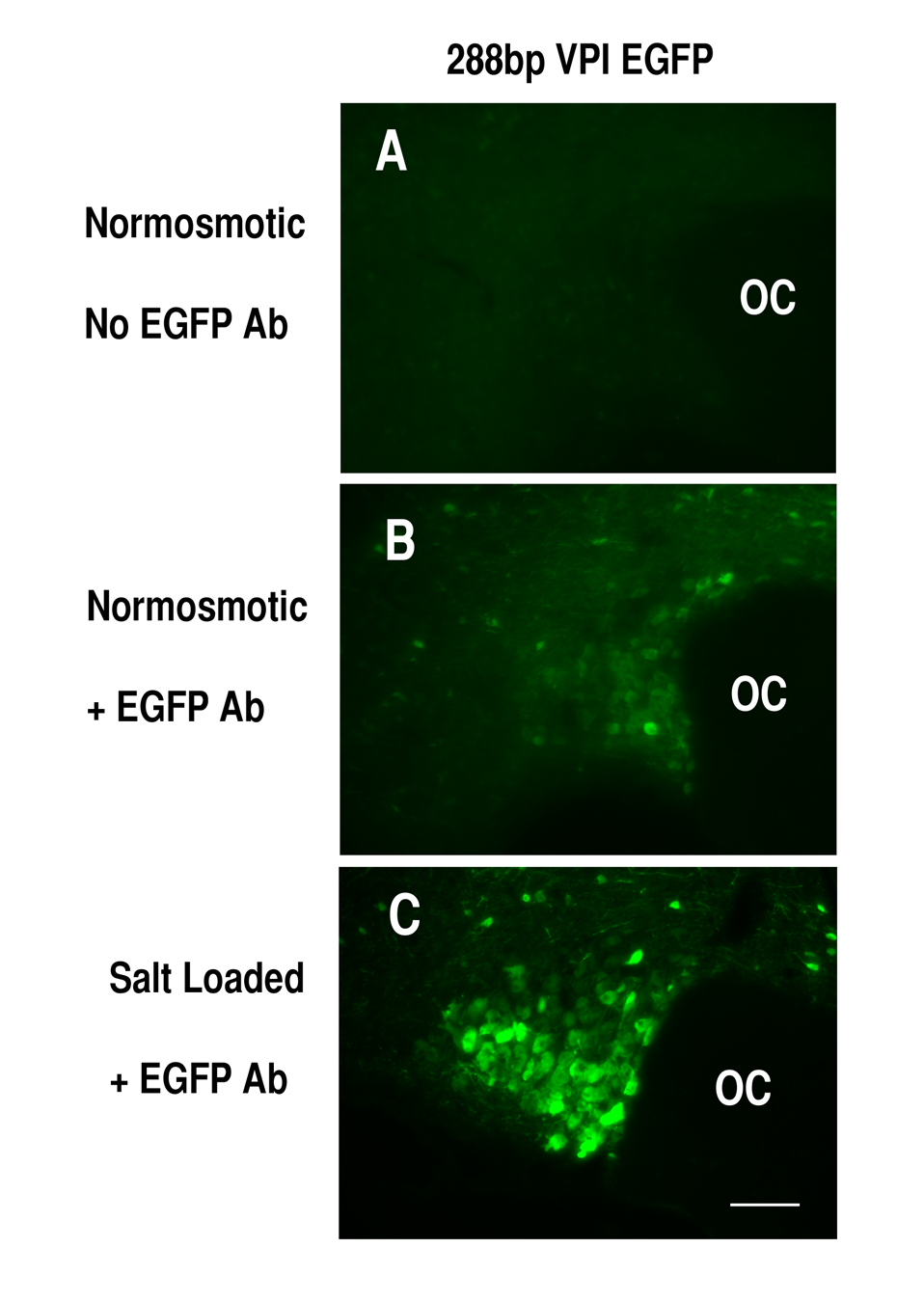

Supplement: Figure S3 — Expression of the injected 288 bp VPI-EGFP construct in the MCNs in the SON is greatly increased during salt loading stimulation. A. Endogenous EGFP fluorescence is not detectable in the SON under normosmotic conditions. B. EGFP expression is detectable in the SON under normosmotic conditions after EGFP IHC. C. EGFP- is strongly expressed in the SON under salt loading conditions with EGFP IHC illustrating that the element responsive to osmotic stimulation is present in the 288 bp promoter construct. 100 µm scale bar in panel C is the same for all images. Abbreviation: OC, optic chiasm. (TIF) [file pone.0048860.s003.tif]

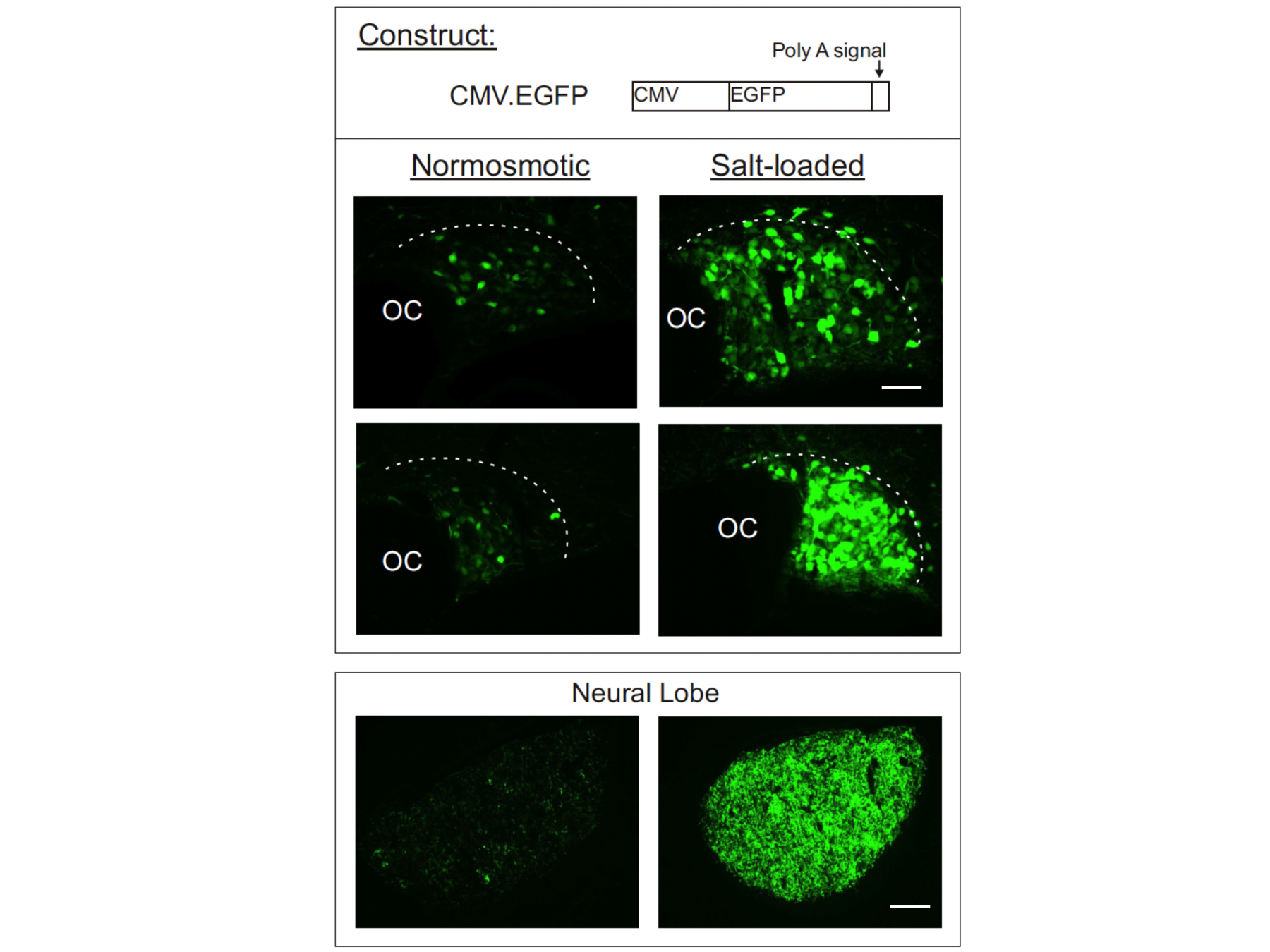

Supplement: Figure S4 — Expression of the cytomegalovirus (CMV) promoter driven expression of EGFP is dramatically increased under salt loading conditions. Rats were injected with the construct shown in the top panel and, after one week, either maintained under control conditions (normosmotic) or subjected to salt loading for an additional week. Middle panels: Endogenous EGFP fluorescence (expression) in salt loaded SONs is much higher than in control (normosmotic) animals as illustrated in two separate rat brain sections. Consistent with this, robust EGFP fluorescence was also observed in the neural lobe of the pituitary gland (bottom panel) with salt loading. Scale bar = 100 µm. Abbreviation: OC, optic chiasm. (TIF) [file pone.0048860.s004.tif]
