# Supplementary material for: Cell-Type Specific Expression of the Vasopressin Gene Analyzed by AAV Mediated Gene Delivery of Promoter Deletion Constructs into the Rat SON In Vivo
Source: PLoS One. 2012 Nov 14;7(11):e48860. doi: 10.1371/journal.pone.0048860 (PMC3498266; doi:10.1371/journal.pone.0048860)
Supplement: Methods S1 — Supplemental Methods. (DOCX) [file pone.0048860.s005.docx]

Supplemental Methods

# Baculovirus and rAAV construction

We used a recombinant Baculovirus (rBV) method that employed insect cells for the production of rAAVs, and which has been shown to result in increased rAAV production per transfected cell as compared to the method using 293T cells [19]. rBVs were made as precursors to rAAV production in Sf9 insect cells using the Bac-to-Bac Baculovirus Expression System according to the manufacturer’s instructions (Invitrogen, Carlsbad CA USA Cat# 10359-016). In order to calculate accurate rBV ratios and Multiplicity of Infections (MOIs) during the production of the rAAVs, rBV titers were determined by qPCR using a procedure modified from Lo and Chao[20].Briefly, we used serial dilutions of intact rBVs as the template for qPCR along with primers targeting the B.V. genome. Primers were designed using the MacVector 7.0 program 7.0 (MacVector, Cary NC, USA) and the *Autographa californica* nucleopolyhedrovirus (BV) DNA sequence (Accession# NC_001623). The primer sequences used were B.V.109103-109124 5'- ATGAAGCACAGTGTCCACGGTC -3' and B.V. 109286-109264 5'- GGTGAAGCGGCAGAATAACAATC -3' which amplify a 184bp amplicon from the viral envelope glycoprotein. The qPCR reaction was performed using a Smart Cycler quantitative PCR system (Cepheid, Sunnyvale CA, USA). The reactions were done using Real-Time SYBR Green PCR master mix (SA Biosciences, Frederick MD USA, Cat# PA-010) according to the manufacturer’s protocol and using 1uL of serially diluted template (1:1k, 1:10k). The run profile was 95^o^C for 15min followed by 40 cycles of 95^o^C for 15s, 60^o^C for 30s, 72^o^C for 30s, and this was followed by a 60^o^C-95^o^C melt curve. To generate a standard curve for rBV titering, DNA was isolated from rBV Bac-RepCap6 according to the method of O’Reilly et al [21], and serial dilutions of the rBV Bac-RepCap6 were used as the control templates. Titers of the Baculovirus DNA were determined by qPCR using the same primers described above on serially diluted samples (1:100 through 1:100k). Titers for all newly manufactured rBV stocks were determined from this standard curve.

In preliminary experiments, we found that of the several rAAV serotypes tested (AAV2, AAV5, AAV6 AAV8, and AAV9), serotype 6 was one of the most efficient in transducing magnocellular neurons in the SON. Therefore, a rBV containing the AAV2 replication gene and the AAV6 capsid gene, Bac-RepCap6, (a gift from Dr. Robert Kotin, NIH/NHLBI) which produces a serotype 6 rAAV was used for all of the rAAV packaging reactions [22]. rBVs containing the Avp constructs shown in Figure 1 were combined with the Bac-RepCap6 rBV at a ratio of 3:1 and used to infect 100 ml of Sf9 cells at density of 2 X 10^6^ cells/ml in suspension culture at a final MOI of 9:3:1 [9(rBV): 3(Bac-RepCap6):1 (Sf9 cell)] according to the method of Negrete et al [23]. Cells were incubated for 3-4 days at 27^o^C while shaking at 115 rpm, until lysis-induced mortality reached approximately 50% as measured by Trypan Blue staining (Fisher Scientific, Pittsburg PA, USA Cat# ICN1691049 ). Cells were harvested by centrifugation at an RCF of 2000 x g for 10 min and the supernatant was saved. The cell pellet was resuspended into 10 ml of sterile PBS and the resuspended cells were subjected to three freeze/thaw cycles, and then centrifuged again at 2000 x g for 10 min. The supernatant from this centrifugation was combined with the first supernatant and MgCl_2_ was added for a final concentration of 2mM. This mixture was then incubated at 37^o^C for 30 min with 2000U of benzonase nuclease (Sigma, St. Louis MO USA, Cat# E1014). After this incubation, PEG -8000 (Sigma, St. Louis, MO USA, Cat# 83271) was added to a final PEG concentration of 2% and the mixture was incubated over night at 4^o^C. The solution was centrifuged at RFC 4000 x g for 20 min to pellet the viral particles and the supernatant was discarded. The rAAV viral pellet was re-suspended in 12ml of a CsCl solution with a refractive index of 1.372. The rAAV was concentrated and purified via a CsCl gradient through centrifugation at 38,000 rpm in a SW Ti-41 rotor (Beckman Coulter, Brea, CA USA) for 48-72hrs. Fractions with a refractive index between 1.378-1.368 were pooled and the CsCl was exchanged for PBS with 2mM MgCl_2_ using an Amicon Ultra-4 spin filter (Millipore, Bedford MA, USA Cat# UFC810024).
